# The effect of tislelizumab on complete and pathological complete response in non-small cell lung cancer: a systematic review and meta-analysis

**DOI:** 10.3389/fonc.2025.1657282

**Published:** 2025-10-08

**Authors:** Qian Feng, Xiaoxia Yan, Liping Gao, Hui Li, Baik Sarah, Russo Anna, Hongying Jiang

**Affiliations:** ^1^ Respiratory Rehabilitation Center, Beijing Rehabilitation Hospital, Beijing, China; ^2^ Department of Pneumology, Lanzhou University Second Hospital, Lanzhou, Gansu, China; ^3^ Department of Biochemistry and Molecular Biology, University of South Alabama, Mobile, AL, United States; ^4^ Medical Oncology and Immunotherapy, Center for Immuno-Oncology, University Hospital of Siena, Siena, Italy

**Keywords:** tislelizumab, NSCLC, complete response, pathological complete response, meta-analysis

## Abstract

**Background:**

Immune checkpoint inhibitors have transformed non-small cell lung cancer (NSCLC) treatment, and while overall survival (OS) and progression-free survival (PFS) are well-established, a comprehensive meta-analysis focusing on complete response (CR) and pathological complete response (pCR) with tislelizumab-based therapies in NSCLC is lacking.

**Methods:**

This systematic review and meta-analysis was conducted following PRISMA guidelines. A thorough literature search was performed across PubMed, Embase, and Web of Science. We included both randomized controlled trials and observational studies of tislelizumab in NSCLC, focusing on extracting data for radiological complete response (CR, based on RECIST 1.1 criteria) and pathological complete response (pCR, defined as absence of residual invasive cancer in resected surgical specimens). Risk of bias was assessed using the Cochrane Collaboration’s tool and the Newcastle-Ottawa Scale. Statistical analyses were performed using the ‘meta’ package in R. 95% confidence intervals (CIs) and odds ratios (ORs) were calculated for CR and pCR, and subgroup analyses were conducted.

**Results:**

7 studies were enrolled in the meta-analysis. The results on pCR showed significant heterogeneity (I^2^ = 92.5%), with a random effects OR of 2.1103 (95% CI: 0.5195 to 8.5727). Subgroup analysis for pCR by disease type revealed a statistically significant difference between NSCLC and SCC only subgroups under the common effect model (p < 0.001). Furthermore, the pCR subgroup analysis by comparator drug showed a statistically significant difference (p < 0.0001) between Pembrolizumab+Chemotherapy (OR 0.6968, 95% CI: 0.3803 to 1.2767) and Chemotherapy alone (OR 7.3123, 95% CI: 2.9204 to 18.3092). For CR, the meta-analysis demonstrated minimal heterogeneity (I^2^ = 0.0%), yielding a significant random effects OR of 2.6277 (95% CI: 1.2858 to 5.3699). Subgroup analysis for CR comparing tislelizumab plus chemotherapy to chemotherapy alone showed a significant advantage (OR 3.8690, 95% CI: 1.5423 to 9.7059).

**Conclusion:**

Tislelizumab combined with chemotherapy significantly improves CR rates in NSCLC compared to chemotherapy alone. While pCR data exhibit high heterogeneity, the findings highlight tislelizumab’s role in achieving deep tumor responses.

## Introduction

Non-small cell lung cancer (NSCLC) is still a dominant cause of cancer-related deaths globally. Immune checkpoint inhibitors (ICIs), especially ICIs targeting PD-1/PD-L1 pathways, have revolutionized the treatment landscape for NSCLC, demonstrating significant clinical benefits and improving patient outcomes. Tislelizumab, a humanized anti-PD-1 monoclonal antibody, has shown promising efficacy and safety in various settings of lung cancer. Previous meta-analyses concerning tislelizumab in lung cancer have primarily focused on classical clinical endpoints such as overall survival (OS) and progression free survival (PFS). For instance, a systematic review and meta-analysis of randomized controlled trials (RCTs) evaluated tislelizumab monotherapy or in combination with chemotherapy for lung cancer, demonstrating significant improvements in both OS (HR: 0.72, 95% CI: 0.63-0.81) and PFS (HR: 0.61, 95% CI: 0.54-0.68) compared to control therapies (1). Another systematic review and network meta-analysis compared tislelizumab with other anti-PD-(L)1 agents in localized advanced or metastatic NSCLC, concluding that tislelizumab, with or without chemotherapy, was comparable or more favorable in terms of OS, PFS, and treatment-related adverse events (2). Similarly, a systematic review of clinical trials on tislelizumab’s safety and effectiveness in NSCLC also highlighted its association with improved PFS and objective response rate (ORR), particularly when combined with chemotherapy (3).

While OS and PFS are crucial long-term indicators of treatment efficacy, comprehensive response assessments, including complete response (CR) and pathological complete response (pCR), offer valuable insights into the immediate and deep tumor responses, especially in the context of neoadjuvant and resectable settings. CR, defined as the absence of detectable tumor, is a significant endpoint in clinical trials and can serve as a surrogate endpoint for accelerated or traditional approval, depending on the disease and context. pCR, defined as the absence of residual invasive and *in situ* cancer in resected specimens after neoadjuvant therapy, is a strong prognostic factor for improved long-term outcomes, including event-free survival (EFS) and OS in various cancers, including NSCLC. Studies have consistently demonstrated that patients achieving pCR after neoadjuvant treatment exhibit significantly better long-term survival rates in NSCLC. Specifically, pCR following neoadjuvant chemo- or chemoradiotherapy has been associated with marked improvements in recurrence and OS. Similarly, rewarding long-term results, including a 5-year OS of 56.18% and disease-free survival of 48.84%, have been observed in locally advanced NSCLC patients who achieve pCR after induction therapy followed by surgery (4). Furthermore, the crucial role of pCR has been highlighted as a surrogate endpoint for predicting and improving clinical outcomes, allowing for shorter duration clinical trials by providing an early indicator of treatment effectiveness (5). The association between neoadjuvant ICI-chemotherapy and meaningful improvement in 2-year EFS and pCR further underscores the importance of this endpoint as a strong surrogate for long-term survival in early-stage NSCLC (6). Thus, increasing the rate of pCR has become a critical endpoint in neoadjuvant trials, with the expectation of translating into improved survival outcomes.

Despite the growing body of evidence supporting the efficacy of tislelizumab in NSCLC, a systematic evaluation of its impact on CR and pCR, integrating data from both RCTs and real-world observational studies, remains an important gap in the literature. Therefore, this meta-analysis aims to specifically address this gap by comprehensively analyzing the available data on CR and pCR rates associated with tislelizumab-based therapies for NSCLC. This study will incorporate evidence from both randomized controlled trials and retrospective/prospective cohort studies to provide a robust assessment of these critical response endpoints.

## Methods

### Search strategy

This systematic review and meta-analysis was conducted in accordance with the Preferred Reporting Items for Systematic Reviews and Meta-Analyses (PRISMA) guidelines (7). A comprehensive literature search was performed across PubMed, Embase, Web of Science databases and Cochrane Library to identify relevant studies. The search strategy combined terms related to lung cancer and tislelizumab, encompassing a broad range of synonyms and classifications. For “lung cancer,” keywords included “lung cancer,” “pulmonary neoplasms,” “bronchial carcinoma,” “lung carcinoma,” and “lung tumor.” For “non-small cell lung cancer,” terms such as “non-small cell lung carcinoma,” “NSCLC,” “adenocarcinoma of lung,” “squamous cell carcinoma of lung,” “large cell lung carcinoma,” “LUAD,” and “LUSC” were utilized. For “tislelizumab,” the term “tislelizumab” was used. The search was performed systematically from inception until the last search date. Additional records were identified through other sources, including screening reference lists of related reviews and enrolled studies. This review was not registered at PROSPERO. The search was performed systematically from inception until May 31, 2025. The full search strings were as follows:

◆ For PubMed: (“lung cancer”[Title/Abstract] OR “pulmonary neoplasms”[MeSH] OR “non-small cell lung cancer”[Title/Abstract] OR “NSCLC”[Title/Abstract]) AND (“tislelizumab”[Title/Abstract] OR “BGB-A317”[Title/Abstract]).
◆ For Embase: ‘lung cancer’:ti,ab,kw AND ‘tislelizumab’:ti,ab,kw.
◆ For Web of Science: TS=(lung cancer OR pulmonary neoplasms OR NSCLC) AND TS=(tislelizumab OR BGB-A317).
◆ For Cochrane Library: (lung cancer OR pulmonary neoplasms OR NSCLC) AND (tislelizumab OR BGB-A317).

Only studies published in English and Chinese were included. Additional records were identified through other sources, including screening reference lists of related reviews and enrolled studies. Furthermore, additional grey literature and ongoing trials were searched via ClinicalTrials.gov, the WHO International Clinical Trials Registry Platform, and conference proceedings to identify relevant unpublished data.

### Eligibility criteria

Studies were included if they investigated tislelizumab, either as monotherapy or in combination with other treatments, for the management of NSCLC. Both RCTs and observational studies (including retrospective and prospective cohort studies) were considered for inclusion. Studies were excluded if they were reviews, case reports, animal studies, meta-analyses, or if they lacked sufficient data for quantitative synthesis or table construction.

### Data and outcome extraction

Two independent reviewers meticulously extracted data from the eligible studies using a standardized data extraction form. Any discrepancies were resolved through discussion or consultation with a third reviewer. For each included study, the following information was extracted: country, study period, study design, total study sample size, patients’ age, proportion of females, specific intervention drugs (including dosage and regimen), and definitions of primary and secondary endpoints. Special emphasis was placed on extracting data pertaining to CR and pCR. According to the RECIST 1.1 criteria, it refers to an objective response observed through imaging, where all target lesions have entirely vanished, no new lesions have appeared, and tumor marker levels have returned to normal. Additionally, within pathological assessment, a pCR is a distinct but related definition, signifying the complete absence of any remaining viable cancer cells in the original tumor area after treatment. Furthermore, detailed information on tumor characteristics, such as specific disease classifications (e.g., squamous vs. non-squamous NSCLC) and PD-L1 expression status, was extracted where available. This detailed extraction aimed to facilitate comprehensive subgroup analyses. Extracted outcomes included radiological complete response (CR, evaluated per RECIST 1.1) and pathological complete response (pCR, assessed in resected specimens).

### Quality assessment

The risk of bias for individual randomized controlled trials was assessed using the Cochrane Collaboration’s risk of bias tool (RoB 2.0) (8). The Newcastle-Ottawa Scale (NOS) was used for observational studies (cohort studies). Publication bias was evaluated using funnel plots, with formal statistical tests such as Egger’s test or Begg’s test used where appropriate, as suggested by methods for quantifying publication bias in meta-analysis (9).

### Statistical analysis

All statistical analyses were performed using the ‘meta’ package in R statistical software (10). For dichotomous outcomes such as pCR and CR, ORs with 95% CIs were calculated. Studies with zero events in both arms were handled by applying a continuity correction of 0.5, as described in Cochrane Handbook. Heterogeneity across studies was assessed using the χ2 test and quantified by the I2 statistic, with I2 values of <25%, 25%−50%, and >50% indicating low, moderate, and high heterogeneity, respectively. A p-value for the χ2 test less than 0.10 or an I2 value greater than 50% was considered to indicate substantial heterogeneity. When heterogeneity was significant, a random effects model (REM) (DerSimonian-Laird method) was applied; otherwise, a fixed effect model (Mantel-Haenszel method for ORs, inverse variance method for continuous data) was used. For studies contributing multiple datasets to the same outcome, we ensured independence by including only one dataset per comparison group in each meta-analysis to avoid double-counting of participants. The random-effects model was consistently applied using the Restricted Maximum-Likelihood (REML) estimator for τ², with confidence intervals calculated via the Q-Profile method. Subgroup analyses were performed based on relevant clinical characteristics, including disease type (e.g., NSCLC vs. SCC only), study design (randomized controlled trials vs. observational studies) and type of comparator drug in the control arm (e.g., Pembrolizumab+Chemotherapy vs. Chemotherapy). Statistical significance was defined as a two-sided p-value < 0.05, unless otherwise specified. Funnel plots were generated to visually inspect for potential publication bias.

## Results

**Figure 1:**
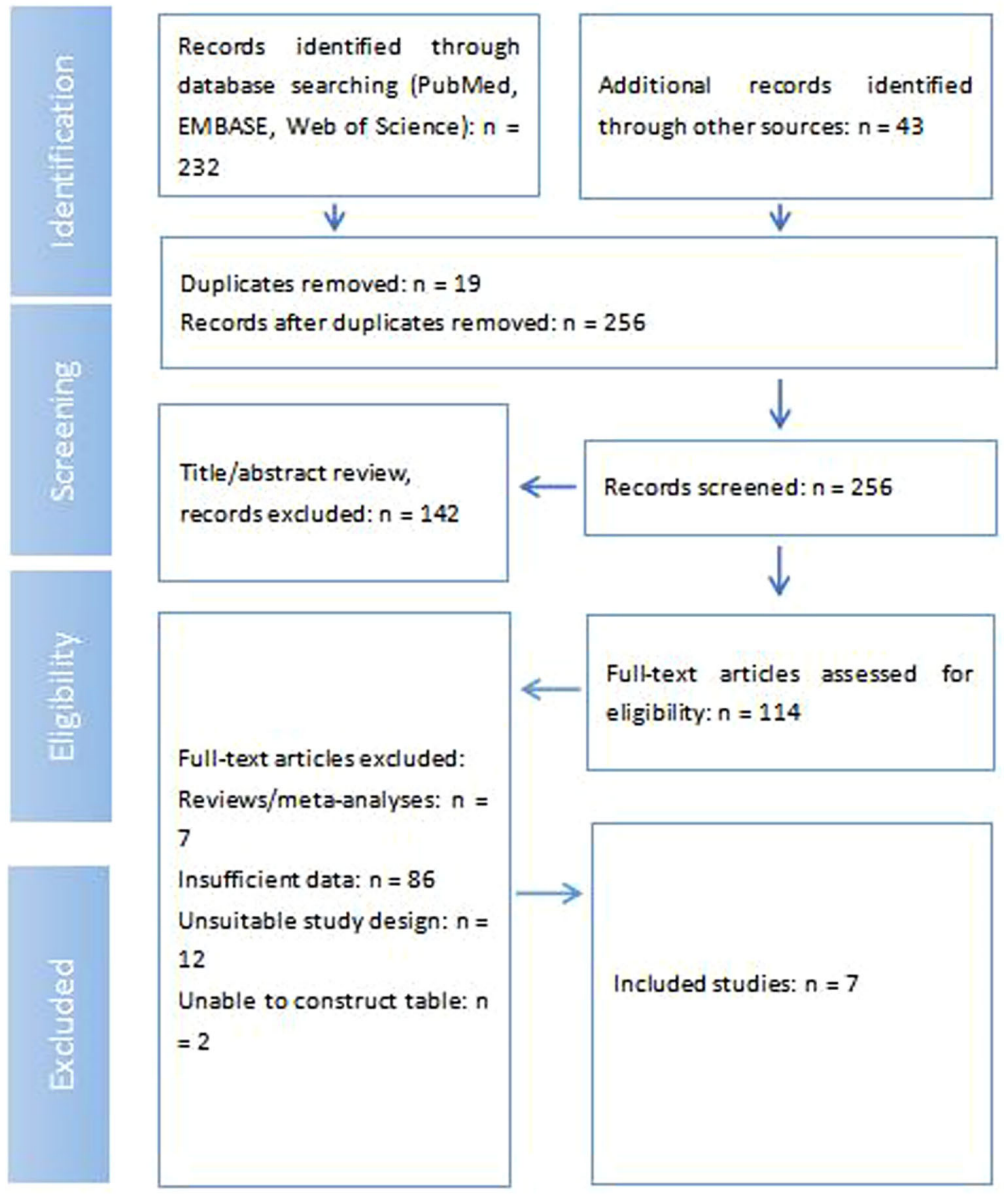
Flow diagram shows the selection process of studies.

232 records in total were initially identified through comprehensive database searching across PubMed, EMBASE, and Web of Science. An additional 43 records were found through other sources, bringing the preliminary total to 275 records. After the removal of 19 duplicate entries, 256 unique records remained for initial screening. All 256 records underwent title and abstract review, which led to the exclusion of 142 records. Consequently, 114 full-text articles were retrieved and subsequently assessed for eligibility. During this full-text review stage, a further 107 articles were excluded for various reasons: 7 were identified as meta-analyses or reviews, 86 lacked sufficient data for inclusion, 12 possessed an unsuitable study design, and 2 could not be formatted into a table. Following this rigorous screening process, a final count of 7 studies met the predefined eligibility criteria, which were ultimately included in this meta-analysis (**Figure 1**).

### Features of enrolled studies on tislelizumab

The enrolled studies for this meta-analysis on Tislelizumab for NSCLC predominantly feature research conducted in China. The study designs vary, encompassing RCTs such as Wang et al., 2024, Yue et al., 2024, and Lu et al., 2024, as well as several retrospective cohort or real-world studies, including Chen et al., 2024, Huang et al., 2024, Yan et al., 2024, and Hu et al., 2025. Patient sample sizes range from 126 (Hu et al., 2025) to 913 (Chen et al., 2024). The age range for eligible patients is generally 18 years and above, with some studies specifying an upper limit of 75–80 years or providing mean ages around 60–64 years. The proportion of females is consistently low across studies where reported, ranging from 7.14% to 18.40%. Intervention drugs typically involve Tislelizumab, often in combination with platinum-based doublet chemotherapy (e.g., paclitaxel, nab-paclitaxel, carboplatin, cisplatin, pemetrexed). Some studies also investigate Tislelizumab alongside other PD-1 inhibitors like pembrolizumab, sintilimab, camrelizumab, nivolumab, and toripalimab, either as monotherapy or in combination with chemotherapy/anti-angiotherapy. Primary endpoints commonly focus on PFS and OS, while some also include EFS and major pathological response (MPR). Notably, pCR is assessed in studies like Huang et al., 2024, and Hu et al., 2025, which focus on neoadjuvant settings. Secondary endpoints are broadly consistent, encompassing ORR, disease control rate (DCR), CR, duration of response (DoR), adverse events (AEs), immune-related AEs, and health-related quality of life (**Table 1**).

**Table 1:** Characteristics of enrolled studies on tislelizumab.

Study	Country	Study period	Study design	Study sample size	Patients age	Proportion of females	Intervention drugs	Primary endpoint	Secondary endpoints
Chen et al., 2024	China	June 2015 to April 2023 (data collection), with the last follow-up on October 11, 2023.	Real-world retrospective study (cohort study)	913 patients	All aged >18 years.	18.40%	PD-1 inhibitors: Pembrolizumab, Sintilimab, Tislelizumab, Camrelizumab, Nivolumab, Toripalimab. Chemotherapy regimens: For non-squamous NSCLC: Pemetrexed (monotherapy or with platinum-based agents like Carboplatin, Cisplatin).For squamous lung cancer: Paclitaxel, Albumin-bound paclitaxel, Gemcitabine, Docetaxel (with or without platinum-based agents).	PFS: Time interval from initiation of first-line treatment to disease progression or death. OS: Time interval from initiation of first-line treatment to death from any cause.	ORR: Sum of PR and CR.DCR
Huang et al., 2024	China	The follow-up period is extended for a minimum of one year after the administration of treatment. The exact start date of patient data collection is not specified in this section, but the approval for the study (Grant No. 2021IIT No. 844) is from 2021.	Retrospective study	154 patients	Mean age 64.23 years (SD 7.79) for the overall cohort. All participants aged 18 years or older.	14.94%	T group: Tislelizumab (200 mg) plus platinum-based dual-drug chemotherapy (for 2–4 cycles pre-surgery).For LUSC: Nab-paclitaxel (260 mg/m2) plus carboplatin (AUC = 5). For LUAD: Pemetrexed (500 mg/m2) plus carboplatin (AUC = 5).| C group: Platinum-based dual-drug chemotherapy alone (for 2–4 cycles pre-surgery). For LUSC: Nab-paclitaxel (260 mg/m2) plus carboplatin (AUC = 5). For LUAD: Pemetrexed (500 mg/m2) plus carboplatin (AUC = 5).	PFS: Defined as the duration from the initiation of treatment until the occurrence of disease progression or mortality due to any cause. OS: Defined as the duration from the initiation of treatment to the date of death resulting from any cause.	the study evaluates Tumor response evaluation based on RECIST 1.1 criteria (CR, PR, SD, PD) and Treatment-related adverse events evaluation. pCR and MPR are also assessed.
Wang et al., 2024	China	Study Start (Actual): 2018-07-30; Primary Completion (Actual): 2020-09-30; Study Completion (Actual): 2023-04-28	Open-label, randomized, multicenter, phase III trial	360 patients	18–75 years	N/A	Arm A: Tislelizumab (200 mg, day 1) + Paclitaxel (175 mg/m2, day 1) + Carboplatin (AUC of 5, day 1);Arm B: Tislelizumab (200 mg, day 1) + Nab-paclitaxel (100 mg/m2, days 1, 8, and 15) + Carboplatin (AUC of 5, day 1);Arm C: Paclitaxel (175 mg/m2, day 1) + Carboplatin (AUC of 5, day 1)	IRC-assessed PFS in arms A and B versus arm C.	OS;IRC and investigator-assessed ORR;IRC- and investigator-assessed DoR; Investigator-assessed PFS; Health-related quality of life;PD-L1 expression as a response biomarker; Safety and tolerability (assessed by monitoring AEs).
Yue et al., 2024	China	Study Start (Actual): 2020-05-29; Primary Completion (Actual): 2023-08-21; Study Completion (Estimated): 2025-10-31	Randomized, double-blind, placebo-controlled phase III trial	450 patients	Eligible patients were aged 18 years or older. The median age is 62–63 years.	N/A	Tislelizumab or placebo, plus platinum-based doublet chemotherapy (cisplatin or carboplatin plus paclitaxel for squamous NSCLC and cisplatin or carboplatin plus pemetrexed for non-squamous NSCLC)	Major pathological response rate and EFS	pCR rate, OS, ORR, disease-free survival, investigator-assessed EFS, health-related quality of life, and safety and tolerability
Yan et al., 2024	China	Between January 2019 and June 2023. Last follow-up and data collection were conducted in November 2023.	Retrospective cohort study	452 patients	18–80 years	18.10%	Pembrolizumab, camrelizumab, tislelizumab, and sintilimab. These were used as ICI monotherapy or combined with chemotherapy/anti-angiotherapy as first-line treatment.	the study assesses tumor response to immunotherapy including ORR, DCR, and PFS.	AEs and Immune-related Adverse Events.
Hu et al., 2025	China	Between December 2017 and August 2023. The last follow-up was set in June 2024.	Single-center retrospective study	126 patients	Aged 18 years and above. The mean age was 59.76 ± 7.05 years.	7.14%	Neoadjuvant pembrolizumab or tislelizumab plus platinum-based doublet chemotherapy.	The study assessed MPR and pCR, DFS and OS.	surgical information, postoperative complications, and toxicity profiles including adverse events and abnormal laboratory findings
Lu et al., 2024	China	From July 23, 2018, through July 31, 2019. Final analysis data cutoff (DCO) was October 26, 2020, and an *ad hoc* updated analysis DCO was July 15, 2022.	Randomized controlled trial	334 eligible patients were randomized. (Tislelizumab plus chemotherapy n=223; chemotherapy alone n=111).	Median age of 62.0 (57.0-67.0) years for the Tislelizumab group and 63.0 (56.0-68.0) years for the Placebo group.	9% (21/226) in the Tislelizumab group and 10% (22/227) in the Placebo group.	Tislelizumab plus chemotherapy or chemotherapy alone. Chemotherapy included cisplatin, carboplatin, and pemetrexed.	PFS assessed by Independent Review Committee (PFSIRC).	OS, antitumor activity including ORR and DoR, and safety and tolerability (including treatment-emergent adverse events) and immune-mediated adverse events.

### Bias analysis of meta-analysis on tislelizumab in NSCLC

**Figure 2:**
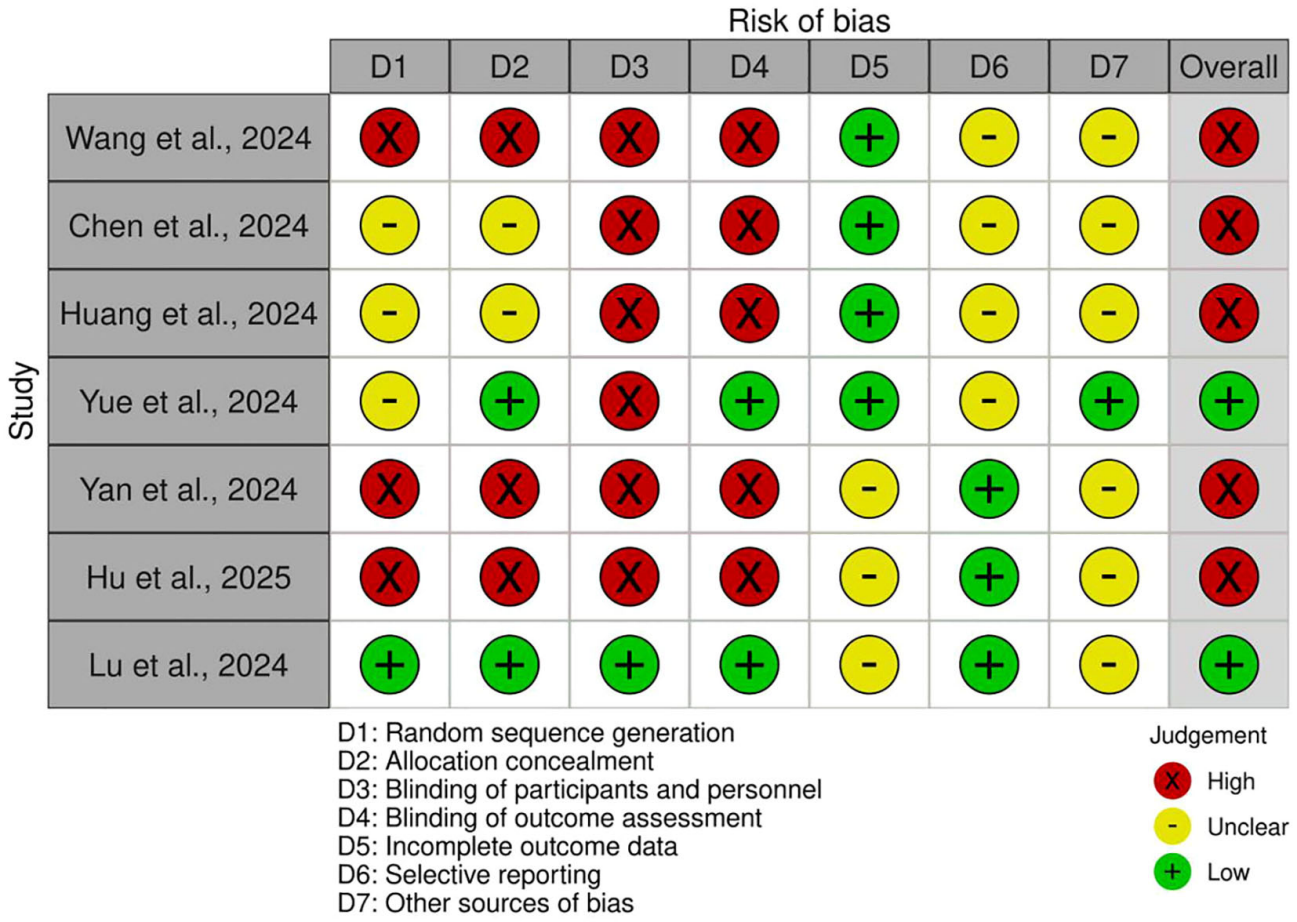
Bias analysis of meta-analysis on tislelizumab in NSCLC.

The bias analysis, shown as **Figure 2**, reveals a mixed picture across the enrolled studies. A substantial portion of the studies demonstrate a high risk of bias, particularly in randomization sequence generation, allocation concealment, and blinding of participants and personnel. Blinding of outcome assessment also frequently presents a high risk. While incomplete outcome data were generally assessed as low risk, other sources of bias and selective reporting often remained unclear. Only one study (Lu et al., 2024) consistently exhibited a low risk of bias in terms of most domains, indicating a higher overall quality. This prevalence of high or unclear bias suggests that the findings of the meta-analysis for tislelizumab in NSCLC should be interpreted with caution, with results potentially influenced by methodological shortcomings in the majority of the included trials.

### Meta-analysis on pCR of tislelizumab plus chemotherapy for NSCLC

**Figure 3:**
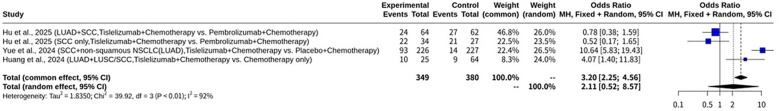
Forest plot of pCR meta-analysis of tislelizumab plus chemotherapy for NSCLC.

**Figure 4:**
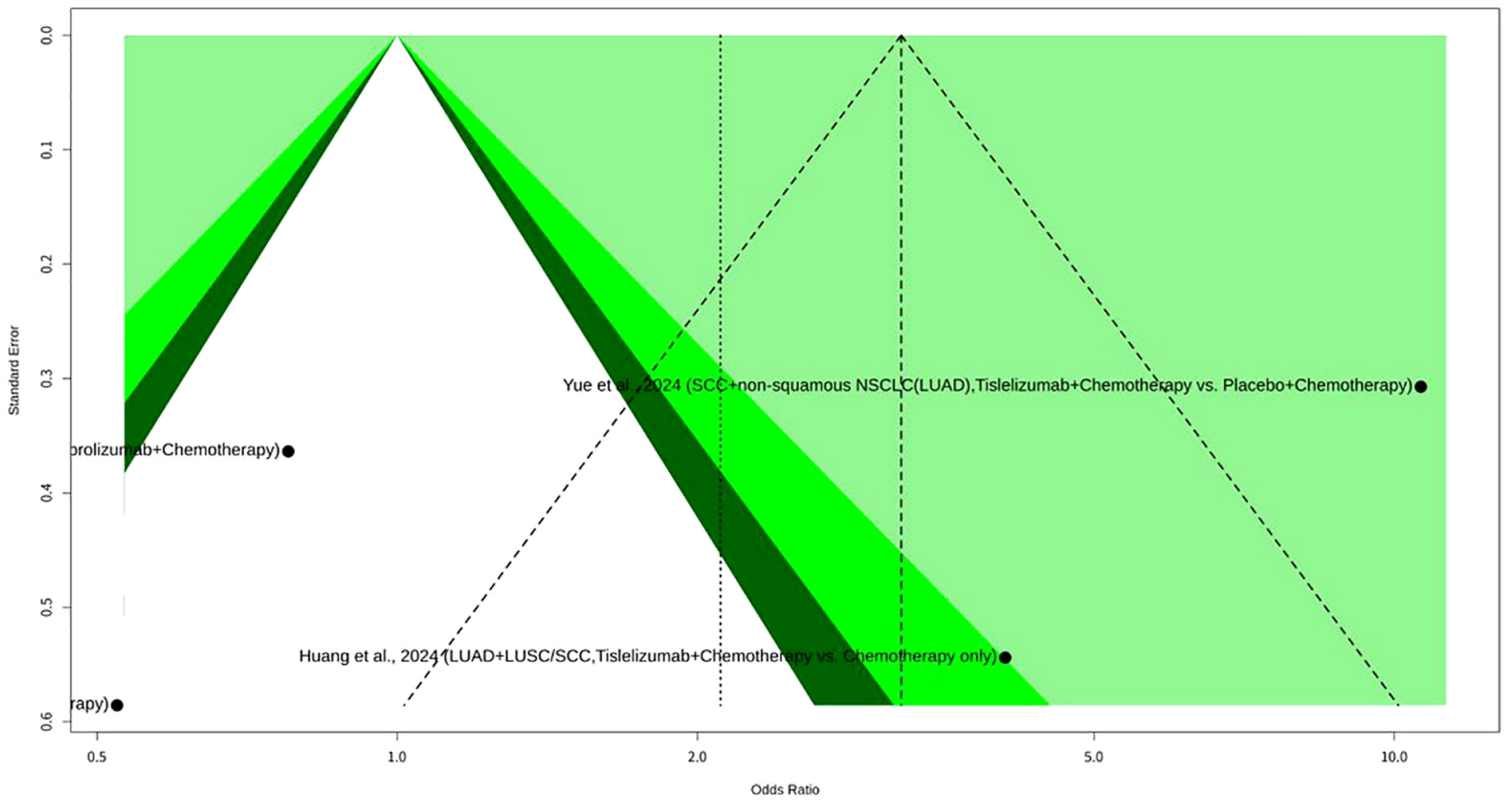
Funnel plot of pCR meta-analysis of tislelizumab plus chemotherapy for NSCLC.

In this meta-analysis on pCR (**Figure 3**) which investigated Tislelizumab plus chemotherapy for NSCLC, a total of four datasets in three studies provided data. The results indicated significant heterogeneity across the enrolled studies, with an I^2^ value of 92.5% (95% CI: 84.0% to 96.5%). The test of heterogeneity yielded a Q statistic of 39.92 with 3 degrees of freedom, resulting in a p-value of less than 0.0001, which strongly indicates substantial heterogeneity. Given this high level of heterogeneity, the REM was deemed more appropriate for evaluating the overall effect size, as it accounts for the variability in treatment effects across different studies. Under the REM, the odds ratio (OR) was 2.1103, with a 95% CI of 0.5195 to 8.5727. The associated Z-value was 1.04, and the p-value was 0.2964. In contrast, the common effect model (CEM), which assumes a single true effect size across all studies, showed an OR of 3.2046 (95% CI: 2.2527 to 4.5586), with a Z-value of 6.48 and a p-value of less than 0.0001. The heterogeneity parameters for the REM included a tau$^2$ of 1.8350 (95% CI: 0.4615 to 27.5376) and a tau of 1.3546 (95% CI: 0.6793 to 5.2476). The meta-analytical method employed the Mantel-Haenszel method and the inverse variance method. The Restricted Maximum-Likelihood estimator was utilized for tau^2^, and the Q-Profile method was used for calculating the CIs of tau^2^ and tau. The enrolled studies contributed various patient cohorts to this meta-analysis. Hu et al., 2025 contributed two sets of data: one for NSCLC (LUAD and SCC combined) with 64 patients in the Tislelizumab plus chemotherapy group (24 events) and 62 patients in the Pembrolizumab plus chemotherapy group (27 events), and another specifically for squamous cell carcinoma (SCC only) with 34 patients in the Tislelizumab plus chemotherapy group (22 events) and 27 patients in the Pembrolizumab plus chemotherapy group (21 events). Yue et al., 2024 investigated NSCLC, including SCC and non-squamous NSCLC (including LUAD), with 226 patients in the Tislelizumab plus chemotherapy group (93 events) and 227 patients in the Placebo plus chemotherapy group (14 events). Lastly, Huang et al., 2024 focused on NSCLC, specifically LUAD and LUSC/SCC, involving 25 patients in the Tislelizumab plus chemotherapy group (10 events) and 64 patients in the chemotherapy-only group (9 events). Funnel plot analysis did not reveal a typical inverted funnel distribution, as shown in **Figure 4**.

**Figure 5:**
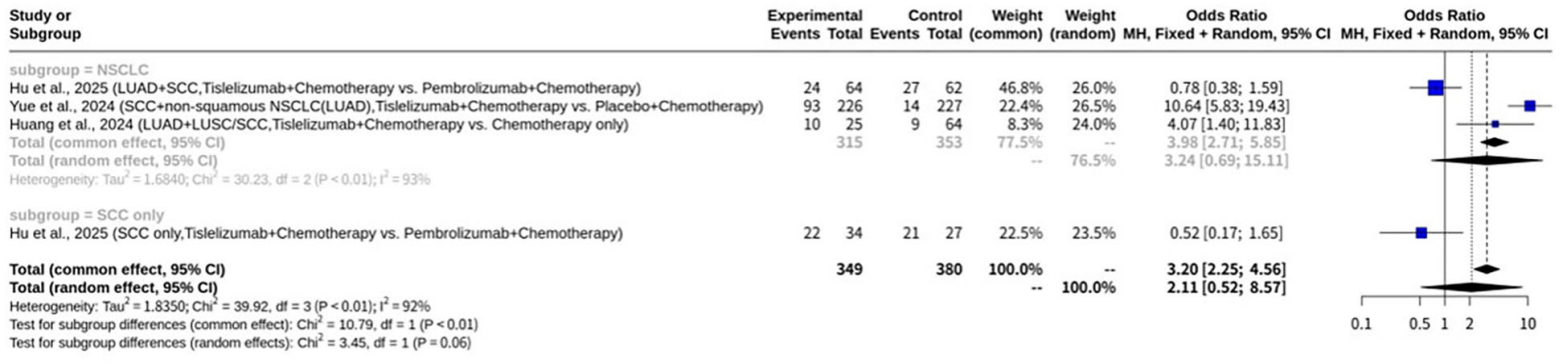
Forest plot of pCR subgroup analysis by disease type for tislelizumab plus chemotherapy in NSCLC.

Subgroup analysis was conducted based on disease type, categorizing studies into NSCLC (encompassing various NSCLC histological types) and SCC only. For the NSCLC subgroup, consisting of 3 studies, the CEM yielded an OR of 3.9845 (95% CI: 2.7134 to 5.8510). Within this subgroup, significant heterogeneity was still present, with a Q statistic of 30.23 and an I^2^ of 93.4%. The REM for the NSCLC subgroup showed an OR of 3.2372 (95% CI: 0.6934 to 15.1136), with a tau^2^ of 1.6840 and a tau of 1.2977. The SCC only subgroup included one study, for which the CEM reported an OR of 0.5238 (95% CI: 0.1662 to 1.6510). As this subgroup comprised only one study, heterogeneity measures were not applicable. Similarly, the REM for the SCC only subgroup also yielded an OR of 0.5238 (95% CI: 0.1662 to 1.6510). A test for subgroup differences using the CEM indicated a statistically significant difference between the NSCLC and SCC only subgroups (Q = 10.79, d.f. = 1, p-value = 0.0010). However, when assessed with the REM, the test for subgroup differences showed a p-value of 0.0632 (Q = 3.45, d.f. = 1), suggesting that the difference in effects between these subgroups was not statistically significant under this model. The results of this subgroup analysis are further illustrated in **Figure 5**.

**Figure 6:**
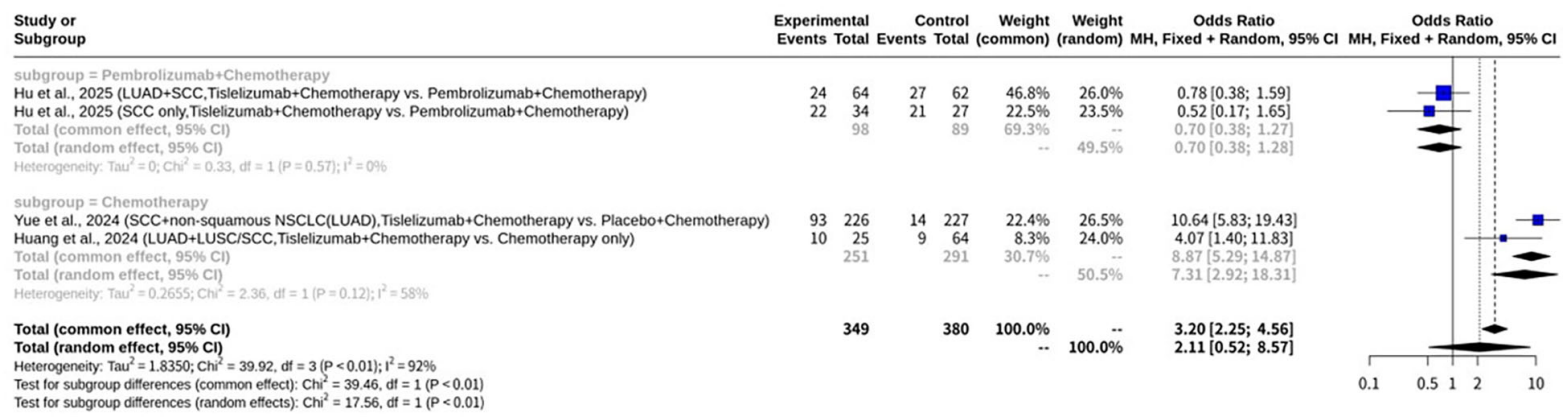
Forest plot of pCR subgroup analysis by comparator drug used in the control arm for tislelizumab plus chemotherapy in NSCLC.

Subgroup analysis was also performed based on the type of comparator drug used in the control arm, creating two subgroups: Pembrolizumab+Chemotherapy and Chemotherapy. For the Pembrolizumab+Chemotherapy subgroup, comprising two studies, the CEM indicated an OR of 0.6952 (95% CI: 0.3802 to 1.2711). There was no heterogeneity observed within this subgroup (Q = 0.33, I^2^ = 0.0%). The REM for this subgroup yielded an OR of 0.6968 (95% CI: 0.3803 to 1.2767), with a tau^2^ of 0. Conversely, for the Chemotherapy subgroup, also consisting of two studies, the CEM showed an OR of 8.8691 (95% CI: 5.2902 to 14.8689). Moderate heterogeneity was present within this subgroup, with a Q statistic of 2.36 and an I^2^ of 57.6%. Under the REM, the Chemotherapy subgroup demonstrated an OR of 7.3123 (95% CI: 2.9204 to 18.3092), with a tau^2^ of 0.2655 and a tau of 0.5152. A test for subgroup differences was conducted. Under the CEM, a highly statistically significant difference was found between the Pembrolizumab+Chemotherapy and Chemotherapy subgroups (Q = 39.46, d.f. = 1, p-value < 0.0001). Similarly, the REM also revealed a statistically significant difference between these subgroups (Q = 17.56, d.f. = 1, p-value < 0.0001). The results of this subgroup analysis are visually represented in **Figure 6**.

### Meta-analysis on complete response of tislelizumab plus chemotherapy for NSCLC

**Figure 7:**
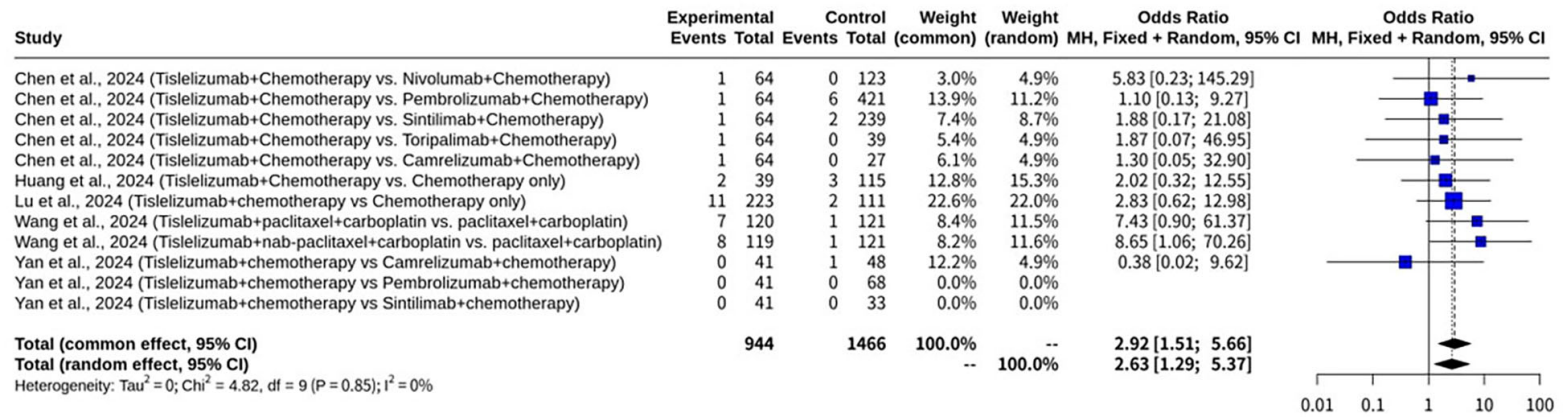
Forest plot of complete response meta-analysis of tislelizumab combined with chemotherapy for NSCLC.

**Figure 8:**
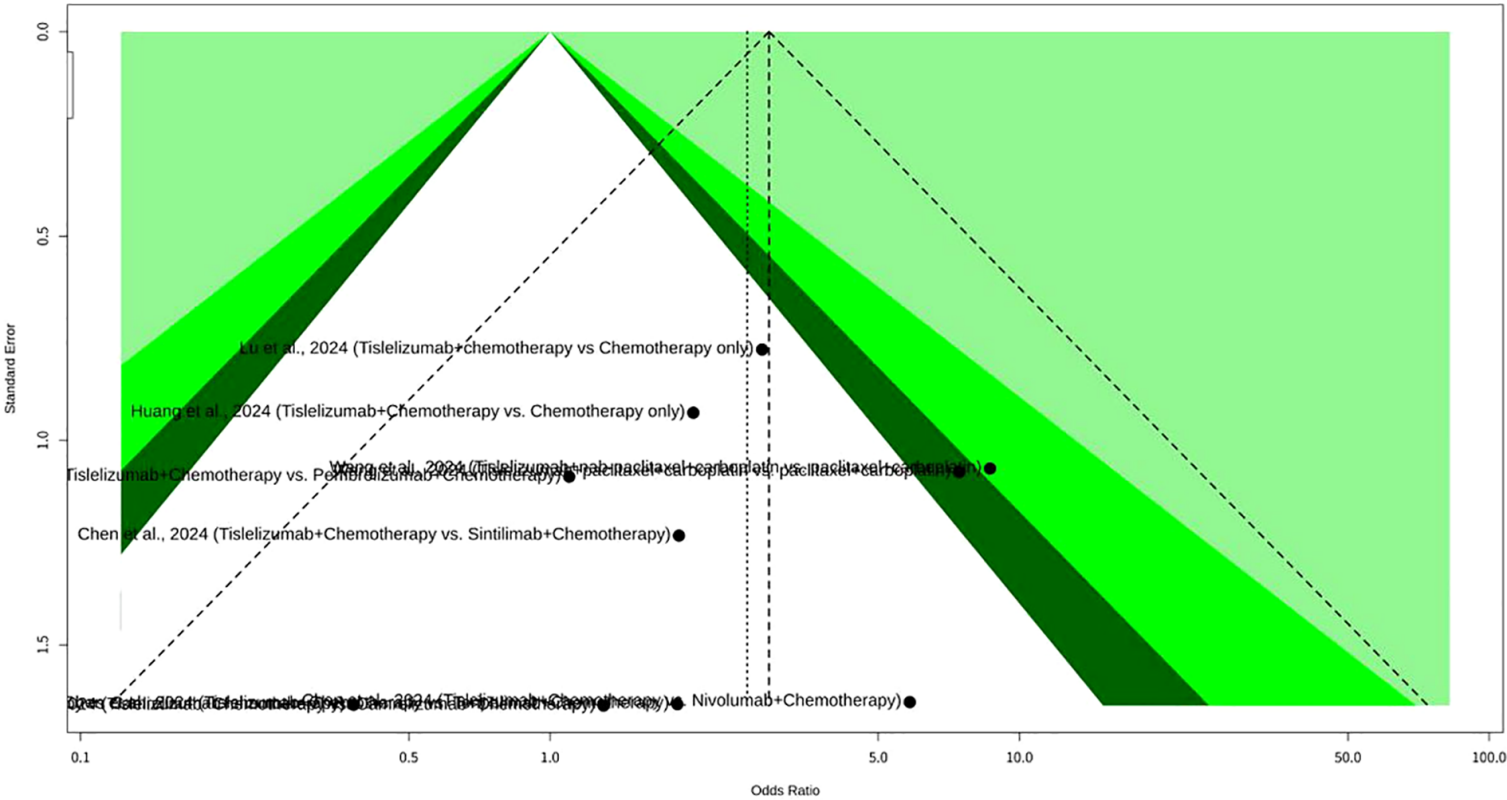
Funnel plot of complete response meta-analysis of tislelizumab combined with chemotherapy for NSCLC.

This meta-analysis, shown as **Figure 7**, investigated the CR rates of Tislelizumab combined with chemotherapy for NSCLC, incorporating data from 10 datasets. Notably, the analysis demonstrated minimal heterogeneity among the enrolled studies. The I^2^ value was 0.0% (95% CI: 0.0% to 62.4%), and the test of heterogeneity yielded a Q statistic of 4.82 with 9 degrees of freedom, resulting in a p-value of 0.8501. This low level of heterogeneity indicates that the treatment effects were highly consistent across the different studies. Given this minimal heterogeneity, both the CEM and the REM provided similar and statistically significant results. Under the CEM, the OR for CR was 2.9249 (95% CI: 1.5114 to 5.6604), with an associated Z-value of 3.19 and a p-value of 0.0014. The REM also demonstrated a statistically significant effect, with an OR of 2.6277 (95% CI: 1.2858 to 5.3699), a Z-value of 2.65, and a p-value of 0.0081. The heterogeneity parameters for the REM showed a tau^2^ of 0 (95% CI: 0.0000 to 1.2253) and a tau of 0 (95% CI: 0.0000 to 1.1069), further confirming the absence of substantial heterogeneity. Funnel plot analysis generally showed an inverted funnel distribution, as depicted in **Figure 8**.

**Figure 9:**
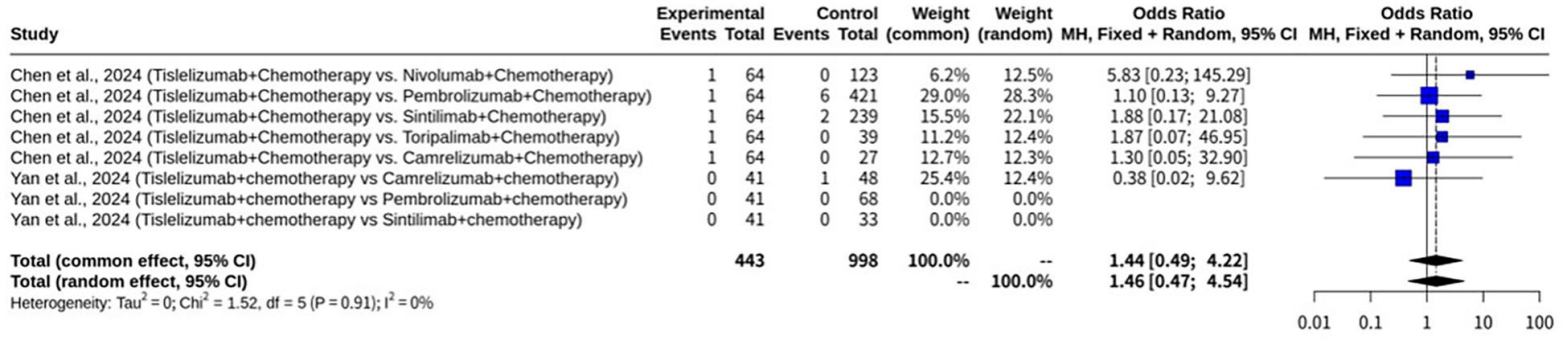
Forest plot of complete response subgroup meta-analysis of tislelizumab combined with chemotherapy for NSCLC focusing on studies where the control arm involved another immunotherapy combined with chemotherapy.

This specific subgroup analysis on CR rates, shown as **Figure 9**, focusing on studies where the control arm involved another immunotherapy combined with chemotherapy, included 8 datasets. Both fixed and REM were applied. Both fixed and random effects models were applied. The analysis revealed minimal heterogeneity among these studies, with an I^2^ value of 0.0% (95% CI: 0.0% to 74.6%) and a non-significant heterogeneity test (Q = 1.52, d.f. = 5, p-value = 0.9113). Under the CEM, the OR for CR was 1.4444 (95% CI: 0.4946 to 4.2182), which was not statistically significant (Z = 0.67, p-value = 0.5014). Similarly, the REM also did not show a statistically significant effect, with an OR of 1.4570 (95% CI: 0.4681 to 4.5353), a Z-value of 0.65, and a p-value of 0.5159. The heterogeneity parameters for the REM further confirmed the low heterogeneity, with a tau^2^ of 0 (95% CI: 0.0000 to 2.1070) and a tau of 0 (95% CI: 0.0000 to 1.4516).

**Figure 10:**
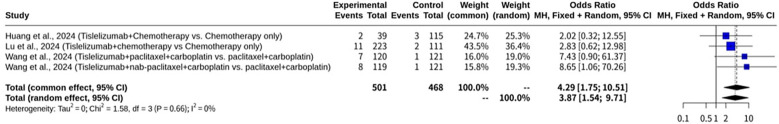
Forest plot of complete response subgroup meta-analysis of tislelizumab combined with chemotherapy for NSCLC focusing on studies where the control arm involved chemotherapy only.

This subgroup analysis on CR rates, shown as **Figure 10**, specifically for studies where the control arm involved chemotherapy alone, included 4 datasets. Both fixed and random effects models were utilized for this analysis. Minimal heterogeneity was observed among these studies, as indicated by an I^2^ value of 0.0% (95% CI: 0.0% to 84.7%) and a non-significant heterogeneity test (Q = 1.58, d.f. = 3, p-value = 0.6631). Under the CEM, Tislelizumab combined with chemotherapy demonstrated a statistically significant advantage in CR, with an OR of 4.2887 (95% CI: 1.7495 to 10.5128), a Z-value of 3.18, and a p-value of 0.0015. The REM also yielded a statistically significant result, showing an OR of 3.8690 (95% CI: 1.5423 to 9.7059), a Z-value of 2.88, and a p-value of 0.0039. The heterogeneity parameters for the REM confirmed low heterogeneity, with a tau^2^ of 0 (95% CI: 0.0000 to 6.1398) and a tau of 0 (95% CI: 0.0000 to 2.4779).

### The safety and tolerability profile of tislelizumab

Emerging clinical data highlight the potential of perioperative tislelizumab to significantly improve outcomes in resectable non-small cell lung cancer. An interim analysis from the phase 3 RATIONALE-315 study demonstrated that perioperative tislelizumab plus neoadjuvant chemotherapy led to a clinically meaningful and statistically significant improvement in both event-free survival and major pathological response rate compared to the placebo group. These findings are supported by smaller, single-center studies that also provide compelling evidence for tislelizumab plus surgery as a viable treatment option for stage II-III NSCLC.

The safety and tolerability profile of tislelizumab is a key consideration for its use in combination with surgery. The RATIONALE-315 interim analysis showed a manageable safety profile, with exposure-adjusted adverse event rates in the tislelizumab arms being lower than in the placebo arm [PMID-39461775]. Moreover, the safety profile has been found to be consistent with prior reports and may even have fewer adverse events compared to classical chemotherapy in some respects. A dedicated analysis of immune-related adverse events (irAEs) across various PD-1 inhibitors, including tislelizumab, found no statistically significant difference in the incidence of irAEs at any grade between the different agents. This indicates a comparable and predictable irAE profile for tislelizumab. Interestingly, this analysis also found a statistically significant association between the occurrence of irAEs and improved progression-free survival and objective response rates.

## Discussion

This meta-analysis provides a comprehensive assessment of the efficacy and safety of tislelizumab for NSCLC, with a focus on pCR and CR rates. In line with the principles of evidence-based medicine, our findings have been systematically evaluated using the GRADE approach to determine the certainty of the evidence for each key outcome. This formal assessment revealed significant variations in the certainty of our findings. The evidence for pathological complete response (pCR) was rated as very low certainty, primarily due to a high risk of bias across the included studies, substantial heterogeneity, and imprecision stemming from wide confidence intervals that included the null effect. This very low certainty underscores the need for extreme caution when interpreting the pooled pCR data. In contrast, the evidence supporting a significant advantage for tislelizumab plus chemotherapy in achieving CR compared to chemotherapy alone was rated as low certainty. This outcome benefited from minimal heterogeneity and a precise effect estimate, but the overall rating was downgraded due to serious limitations in the study designs, including the prevalence of non-randomized and high-bias studies. Lastly, the evidence for the comparison of tislelizumab plus chemotherapy against other immunotherapy plus chemotherapy regimens for CR was also of very low certainty, a result of both serious study limitations and a wide, imprecise confidence interval that failed to demonstrate a statistically significant difference. These nuanced certainty ratings are critical for contextualizing our results and guiding the subsequent discussion of potential contributing factors and study limitations (**Table 2**).

**Table 2:** GRADE assessment of evidence certainty.

Outcome	Study limitations (Risk of Bias)	Inconsistency (Heterogeneity)	Indirectness	Imprecision	Publication bias	Certainty of evidence	Justification
pCR (Tislelizumab + Chemo vs. Control)	Serious. Majority of studies had high/unclear risk of bias in key domains (randomization, allocation concealment, blinding).	Very Serious. Extremely high heterogeneity (I2 = 92.5%, p<0.0001). The substantial variability in results cannot be fully explained by subgroup analyses.	Not serious. Studies directly address the research question.	Serious. The 95% CI is wide and includes the null effect (OR 0.52 to 8.57), suggesting a large degree of uncertainty.	Undetected. Funnel plot analysis did not reveal a typical inverted funnel plot.	Very Low.	Downgraded due to a high risk of bias, extreme heterogeneity, and wide confidence intervals.
CR (Tislelizumab + Chemo vs. Chemo Alone)	Serious. Based on the overall assessment of included studies, which contained many non-RCTs and had a high risk of bias.	Not serious. Minimal heterogeneity observed (I2 = 0.0%, p=0.6631).	Not serious. Studies directly compare the interventions of interest.	Not serious. The 95% CI is relatively narrow and does not cross the null effect (OR 1.75 to 10.51), indicating a precise and statistically significant effect.	Not applicable. The funnel plot showed an inverted funnel shape, but with only 4 studies, visual inspection is not reliable.	Low.	Downgraded due to serious study limitations (risk of bias) stemming from the prevalence of high-bias studies.
CR (Tislelizumab + Chemo vs. Other IO + Chemo)	Serious. Based on the overall assessment of included studies, which contained many non-RCTs and had a high risk of bias.	Not serious. Minimal heterogeneity observed (I2 = 0.0%, p=0.9113).	Not serious. Studies directly compare the interventions of interest.	Serious. The 95% CI is wide and includes the null effect (OR 0.49 to 4.22), suggesting a high degree of imprecision and no statistically significant effect.	Undetected. Funnel plot shape is difficult to interpret with limited studies.	Very Low.	Downgraded due to serious study limitations (risk of bias) and imprecision (wide confidence interval including the null).

The meta-analysis on pCR rates for Tislelizumab plus chemotherapy in NSCLC revealed substantial heterogeneity, indicating considerable variability in treatment effects across the enrolled studies. Additionally, the lack of standardization in pathological assessment across studies, including variability in the number of sections examined, sampling thoroughness, and pathologist experience, may have contributed to the observed heterogeneity in pCR rates. Differences in pathological review protocols could introduce unmeasured variability in pCR determination, potentially influencing reported outcomes. Future studies should adhere to standardized pathological assessment guidelines, such as those proposed by the International Association for the Study of Lung Cancer (IASLC), to minimize such heterogeneity and improve comparability across trials. This high degree of heterogeneity necessitates a thorough discussion of potential contributing factors. One significant source of heterogeneity likely stems from differences in patient populations and disease characteristics, particularly the histological subtypes of NSCLC. While our meta-analysis provided a subgroup analysis for NSCLC (encompassing various histological types) versus SCC only, the broader NSCLC group itself still exhibited high heterogeneity (I^2^ = 93.4%). This suggests that even within the NSCLC category, variations in the precise proportions of LUAD versus squamous cell carcinoma (SCC/LUSC) among studies, or the inclusion of other less common NSCLC subtypes, could impact pCR rates differently. For instance, the Huang et al., 2024 study included both LUAD and LUSC/SCC patients, while Yue et al., 2024 included SCC and non-squamous NSCLC (LUAD). The intrinsic biological differences between these subtypes, including their unique molecular pathways and responsiveness to specific therapeutic agents, could lead to divergent pCR outcomes. Furthermore, the meta-analysis did not provide a detailed breakdown of disease stage within NSCLC. While all enrolled studies focused on resectable stage II-IIIA/IIIb NSCLC, subtle differences in the distribution of specific substages (e.g., II vs. IIIA/B) across studies could influence resectability and pathological response, thereby contributing to heterogeneity. Another critical factor contributing to heterogeneity is the diversity in control group interventions. Our subgroup analysis by comparator drug type highlighted a statistically significant difference in effects between studies using Pembrolizumab+Chemotherapy as a comparator versus those using Chemotherapy alone. Studies like Hu et al., 2025, compared Tislelizumab+Chemotherapy against Pembrolizumab+Chemotherapy, introducing another active immune checkpoint inhibitor in the control arm. In contrast, Huang et al., 2024, compared Tislelizumab+Chemotherapy to chemotherapy alone. The inclusion of another immunotherapy agent in the control arm (Pembrolizumab) inherently provides a more robust comparison against a highly effective regimen, potentially narrowing the observed treatment effect of Tislelizumab, or even showing a non-significant difference as seen in the Pembrolizumab+Chemotherapy subgroup (OR 0.6968, 95% CI: 0.3803 to 1.2767). Conversely, comparing Tislelizumab+Chemotherapy to chemotherapy alone (as in Huang et al., 2024, and Yue et al., 2024 (placebo+chemotherapy)) is likely to demonstrate a more pronounced advantage for the immunotherapy-containing arm, which is reflected in the much higher OR observed in the Chemotherapy subgroup (OR 7.3123, 95% CI: 2.9204 to 18.3092). This fundamental difference in control arm design is a major driver of the observed overall heterogeneity. Beyond the type of comparator drug, the specific chemotherapy regimens themselves, which were not finely stratified in this meta-analysis, could also contribute to variability. Although all studies generally employed platinum-based doublet chemotherapy, the choice of platinum agent (cisplatin vs. carboplatin) and the companion cytotoxic drug (e.g., paclitaxel, pemetrexed, nab-paclitaxel) can influence response rates. For instance, Yue et al., 2024, specified cisplatin or carboplatin plus paclitaxel for squamous NSCLC and cisplatin or carboplatin plus pemetrexed for non-squamous NSCLC. Similarly, Huang et al., 2024, used nab-paclitaxel plus carboplatin for LUSC and pemetrexed plus carboplatin for LUAD. Even subtle differences in chemotherapy dosages, administration schedules, or the number of neoadjuvant cycles (e.g., 2–4 cycles in Huang et al., 2024; 3–4 cycles in Yue et al., 2024) across studies could affect the overall pCR. Finally, unmeasured or unaddressed factors, often categorized as other variables, might also contribute to the high heterogeneity. These could include variations in patient characteristics such as PD-L1 expression levels (though some studies like Yue et al., 2024, stratified by this), genetic mutations (e.g., EGFR/ALK status, though excluded in some studies), baseline tumor burden, patient comorbidities, and differences in surgical techniques or post-operative management protocols across the different clinical centers and regions. Furthermore, variability in surgical techniques (e.g., open vs. minimally invasive approaches), completeness of resection (R0 vs. R1/R2), and the interval between neoadjuvant therapy and surgery may contribute to unmeasured heterogeneity in pCR rates across studies. For instance, longer intervals between neoadjuvant therapy and surgery have been associated with higher pCR rates in some NSCLC studies, while variations in surgical quality and perioperative management may independently influence pathological outcomes. Although these factors were not systematically reported in the enrolled studies, they represent important sources of heterogeneity that should be addressed in future prospective trials through standardized surgical and pathological reporting protocols. Methodological differences, such as retrospective versus prospective study designs (e.g., Hu et al., 2025 and Huang et al., 2024 were retrospective, while Yue et al., 2024 was a randomized, double-blind, placebo-controlled phase 3 trial), could also introduce variability in reported outcomes. The inherent differences in study design and execution further complicate the interpretation of pooled results and contribute to the high I^2^ value observed.

This subgroup meta-analysis specifically examined the CR rates of Tislelizumab combined with chemotherapy compared to other immunotherapy agents combined with chemotherapy in NSCLC patients. The analysis included 6 datasets from two studies: Chen et al., 2024, and Yan et al., 2024. These studies involved comparisons against Nivolumab, Pembrolizumab, Sintilimab, Toripalimab, and Camrelizumab, all combined with chemotherapy. The I^2^ value was 0.0%, and the test for heterogeneity yielded a Q statistic of 1.52 with 5 degrees of freedom, resulting in a non-significant p-value of 0.9113. This indicates a high degree of consistency in the observed effects across the studies. However, despite the consistency, the meta-analysis did not demonstrate a statistically significant difference in CR rates when Tislelizumab plus chemotherapy was compared to other immunotherapy plus chemotherapy regimens. Under the CEM, the OR for CR was 1.4444, which was not statistically significant. Similarly, the REM also did not show a statistically significant effect, with an OR of 1.4570, a Z-value of 0.65, and a p-value of 0.5159. The heterogeneity parameters and tau = 0 further supported the low heterogeneity, meaning that the lack of significance was not attributable to substantial variability between studies. These findings suggest that, in terms of achieving CR, Tislelizumab combined with chemotherapy appears to have comparable efficacy to other PD-1 inhibitors (Nivolumab, Pembrolizumab, Sintilimab, Toripalimab, and Camrelizumab) when each is combined with chemotherapy as first-line treatment for advanced NSCLC. The studies included in this analysis, such as Chen et al., 2024, and Yan et al., 2024, represent real-world clinical practice in China, where these agents are commonly used. While some preclinical studies and network meta-analyses might suggest subtle differences in binding affinities or pharmacokinetics among PD-1 inhibitors, this specific meta-analysis on CR in combination with chemotherapy does not indicate a distinct advantage for Tislelizumab over the other immunotherapies in this outcome. The results align with broader real-world evidence often showing comparable efficacy profiles across various PD-1 inhibitors in similar clinical settings for NSCLC, particularly when combined with chemotherapy.

This subgroup analysis specifically investigated CR rates in studies where Tislelizumab combined with chemotherapy was compared against chemotherapy alone. Both fixed and random effects models were employed, and the results consistently indicated minimal heterogeneity among these studies, with an I^2^ value of 0.0% and a non-significant heterogeneity test. This low heterogeneity suggests that the effects observed were consistent across the different studies within this subgroup. Under the CEM, Tislelizumab combined with chemotherapy demonstrated a statistically significant advantage in achieving CR, with an OR of 4.2887. This finding was supported by a Z-value of 3.18 and a p-value of 0.0015. The REM similarly yielded a statistically significant result, showing an OR of 3.8690, with a Z-value of 2.88 and a p-value of 0.0039. The heterogeneity parameters for the REM, with a tau^2^ of 0 and a tau of 0, further reinforced the conclusion of minimal heterogeneity. The enrolled studies in this subgroup were Huang et al., 2024, Lu et al., 2024, and Wang et al., 2024 (contributing two separate comparisons: Tislelizumab plus paclitaxel plus carboplatin versus paclitaxel plus carboplatin, and Tislelizumab plus nab-paclitaxel plus carboplatin versus paclitaxel plus carboplatin). These results strongly suggest that the addition of Tislelizumab to chemotherapy significantly improves CR rates when compared to chemotherapy alone in NSCLC patients.

This meta-analysis, incorporating both RCTs and real-world retrospective cohort studies from China, provides a comprehensive overview of tislelizumab’s efficacy in NSCLC. Our findings regarding pCR demonstrate significant heterogeneity across the enrolled studies, with a high I^2^ value of 92.5%. Despite this heterogeneity, when examining the pCR of tislelizumab combined with chemotherapy, our REM yielded an OR of 2.1103. This is further supported by individual study data, such as the single-arm study where 76% (35 of 46 patients) of the ITT population achieved MPR, and 52% (24 of 46 patients) achieved pCR in the ITT population, significantly exceeding the predefined threshold of 30% (10). The rate of complete surgical resection (R0) was also notably high in this single-arm study, achieved in 96% (44 of 46) of patients (11). These individual study outcomes lend qualitative support to the potential for favorable pathological responses with tislelizumab-based regimens.

Our subgroup analysis for pCR by disease type revealed a statistically significant difference between NSCLC and SCC only subgroups under the CEM (p-value = 0.0010). However, this difference was not statistically significant under the REM (p-value = 0.0632). This suggests that while there might be a trend, further high-quality studies, especially in SCLC populations, are needed to confirm differential benefits as also highlighted by a recent systematic review.

Regarding CR, our meta-analysis demonstrated consistent and statistically significant benefits of tislelizumab combined with chemotherapy, with minimal heterogeneity across the enrolled studies. The overall OR for CR was 2.6277 under the REM. This positive outcome aligns with findings from another systematic review and meta-analysis which indicated that tislelizumab significantly improved OS and PFS in lung cancer patients. This review, encompassing four Phase III RCTs with 1,837 patients, reported consistent benefits across NSCLC and SCLC populations, and similar efficacy for tislelizumab as monotherapy or in combination with chemotherapy.

While the efficacy data are encouraging, it is crucial to consider the safety profile and potential adverse events associated with tislelizumab. Our enrolled studies examine AEs and immune-related adverse events (irAEs) as secondary endpoints. The interim analysis from the phase 3 RATIONALE-315 study supports a manageable safety profile for tislelizumab when used in combination with neoadjuvant chemotherapy, with exposure-adjusted adverse event rates in the tislelizumab arms being lower than those in the placebo arm [PMID-39461775]. This is consistent with prior reports, suggesting that tislelizumab may have a favorable safety profile compared to classical chemotherapy in certain aspects. Furthermore, a dedicated analysis of immune-related adverse events (irAEs) across various PD-1 inhibitors, including tislelizumab, revealed no statistically significant difference in the incidence of irAEs at any grade among the agents, indicating a comparable and predictable irAE profile for tislelizumab. Notably, this analysis also identified a statistically significant association between the occurrence of irAEs and improved progression-free survival and objective response rates, suggesting that the presence of manageable irAEs may serve as a potential biomarker for treatment efficacy. Case reports further highlight the importance of vigilance when using ICIs. For instance, a case of pulmonary artery pseudoaneurysm leading to hemoptysis was reported in a 65-year-old male with squamous cell carcinoma after receiving chemotherapy combined with tislelizumab. Another case documented a 71-year-old male with extensive-stage small cell lung cancer developing type 1 diabetic ketoacidosis (new-onset) induced by tislelizumab after three cycles of treatment, despite no prior history of diabetes (12). These case reports underscore the potential for rare but serious irAEs, emphasizing the necessity of routine glucose monitoring and early recognition of high blood sugar and C-peptide deficiency during ICI treatment to prevent life-threatening endocrine irAEs (13). The overall bias analysis revealed a high or unclear risk of bias in a majority of our enrolled studies, especially in areas of randomization, allocation concealment, and blinding. This underscores the need for continued high-quality RCTs to validate these findings, particularly in specific subgroups, and to better characterize patient-specific factors such as age, gender, and comorbidities for optimized treatment strategies.

### Limitations

Several limitations should be considered when interpreting the results of this meta-analysis. Firstly, the generalizability and external validity of our findings are limited as all included studies were conducted in China, introducing a geographical and ethnic homogeneity. Furthermore, the varying study designs, which predominantly included retrospective cohorts with a high or unclear risk of bias as noted in our bias analysis, restrict the reliability of our conclusions. Secondly, the high heterogeneity observed in the pCR meta-analysis, while partially addressed by subgroup analyses, suggests underlying variations that could not be fully accounted for. These include unmeasured factors such as the detailed molecular characteristics of tumors (beyond broad histological types and PD-L1 status), specific details of prior treatments, or patient-specific comorbidities that were not consistently reported across all studies. Thirdly, the varying study designs, encompassing both RCTs and real-world retrospective cohort studies, introduce inherent differences in methodology and potential for bias. While efforts were made to assess the quality of enrolled studies, the prevalence of high or unclear risk of bias in several trials, particularly concerning randomization, allocation concealment, and blinding, as noted in our bias analysis, necessitates cautious interpretation of the findings. Fourthly, while pCR and CR are highly relevant endpoints, particularly in neoadjuvant settings, they are surrogate markers for long-term survival. Due to the limited mature survival data available across the included studies, this meta-analysis could not robustly examine the correlation between these early responses and long-term outcomes, such as OS or PFS. Finally, the absence of head-to-head comparisons for tislelizumab against all other PD-1/PD-L1 inhibitors in certain clinical settings, as well as the limited number of studies contributing to some subgroups, restrict the robustness of direct comparisons and the ability to draw definitive conclusions regarding superiority or inferiority among different ICIs for specific endpoints.

## Conclusion

This meta-analysis provides compelling evidence that tislelizumab, particularly when combined with chemotherapy, significantly improves CR rates in NSCLC patients compared to chemotherapy alone, with a high degree of consistency across studies. However, it is important to acknowledge that radiological CR serves as a valuable short-term efficacy marker but is not a validated surrogate endpoint for long-term survival in NSCLC. In contrast, pCR has been established as a validated surrogate for long-term survival, particularly in the neoadjuvant setting. While the impact on pCR demonstrates higher heterogeneity, individual study data highlight tislelizumab’s potential for favorable pathological outcomes. In comparisons against other immunotherapy-plus-chemotherapy regimens, tislelizumab shows comparable efficacy in achieving CR. Furthermore, existing pharmacoeconomic evaluations predominantly from China suggest that tislelizumab represents a cost-effective treatment option in various cancer settings, including NSCLC. These findings underscore the clinical utility and economic value of tislelizumab in the management of NSCLC, though the generalizability of these findings is limited by the predominance of data from the Chinese healthcare landscape and the inherent biases and heterogeneity of the included studies. To address these limitations, future high-quality randomized controlled trials with diverse patient populations and standardized reporting are warranted. These studies are crucial to better confirm these benefits globally, explore nuanced differences in response across specific NSCLC subtypes, and further refine optimal treatment strategies.

## Data Availability

The original contributions presented in the study are included in the article/supplementary material. Further inquiries can be directed to the corresponding author.
